# World society of emergency surgery study group initiative on Timing of Acute Care Surgery classification (TACS)

**DOI:** 10.1186/1749-7922-8-17

**Published:** 2013-05-01

**Authors:** Yoram Kluger, Offir Ben-Ishay, Massimo Sartelli, Luca Ansaloni, Ashraf E Abbas, Ferdinando Agresta, Walter L Biffl, Luca Baiocchi, Miklosh Bala, Fausto Catena, Raul Coimbra, Yunfeng Cui, Salomone Di Saverio, Koray Das, Tamer El Zalabany, Gustavo P Fraga, Carlos Augusto Gomes, Ricardo Alessandro Teixeira Gonsaga, Jakub Kenig, Ari Leppäniemi, Sanjay Marwah, Gerson Alves Pereira Junior, Boris Sakakushev, Boonying Siribumrungwong, Norio Sato, Cristian Tranà, Nereo Vettoretto, Ernest E Moore

**Affiliations:** 1 Department of Surgery, Rambam Health Care Center, Haifa, Israel; 2 Department of Surgery, Macerata Hospital, Macerata, Italy; 3 Department of General Surgery, Ospedali Riuniti, Italy; 4 Department of General Surgery, Mansoura University Hospital, Mansoura, Egypt; 5 Department of General Surgery, Department of Surgery, Adria Hospital, Adria, Italy; 6 Denver Health Medical Center, Denver, Colorado, USA; 7 Department of Medical and Surgical Sciences, Surgical Clinic, Brescia University, Brescia, Italy; 8 Department of General Surgery, Hadassah Medical Center, Jerusalem, Israel; 9 Department of Surgery, Division of Trauma, Surgical Critical Care, and Burns, University of California, San Diego Health System, San Diego, California, USA; 10 Department of Surgery, Tianjin Nankai Hospital, Nankai Clinical School of Medicine, Tianjin Medical University, Tianjin, China; 11 Department of Emergency and Trauma Surgery, Maggiore Hospital, Trauma Center, Bologna, Italy; 12 Department of General Surgery, Numune Training and Research Hospital, Adana, Turkey; 13 Department of General Surgery, Bahrain Defence Force Military Hospital, West Riffa, Bahrain; 14 Department of Surgery, University of Campinas, Campinas, São Paulo, Brazil; 15 Department of Surgery, Hospital Universitário, Universidade Federal de Juiz de Fora, Juiz de Fora, Minas Gerais, Brazil; 16 Department Chirurgia General e do Trauma, Catanduva, Brazil; 17 3rd Department of General Surgery, Jagiellonian Univeristy, Narutowicz Hospital, Krakow, Poland; 18 Department of Abdominal Surgery, University Hospital Meilahti, Helsinki, Finland; 19 Department of Surgery, Post- Graduate Institute of Medical Science, Rohatak, India; 20 Emergency Unit, Department of Surgery, Ribeirão Preto, Brazil; 21 First clinic of General Surgery, University Hospital Plovdiv, Plovdiv, Bulgaria; 22 Department of General Surgery, Thammsat University Hospital, Pathumthani, Thailand; 23 Department of Primary care and Emergency medicine, Kyoto University, Kyoto, Japan; 24 Department of Surgery, Ancona University, Ancona, Italy; 25 Department of Surgery, Mellini Hospital, Chiari, Italy; 26 Department of Surgery, Denver Health Medical Center, Denver, Colorado, USA

## Abstract

Timing of surgical intervention is critical for outcomes of patients diagnosed with surgical emergencies. Facing the challenge of multiple patients requiring emergency surgery, or of limited resource availability, the acute care surgeon must triage patients according to their disease process and physiological state. Emergency operations from all surgical disciplines should be scheduled by an agreed time frame that is based on accumulated data of outcomes related to time elapsed from diagnosis to surgery. Although literature exists regarding the optimal timing of various surgical interventions, implementation of protocols for triage of surgical emergencies is lacking. For institutions of a repetitive triage mechanism, further discussion on optimal timing of surgery in diverse surgical emergencies should be encouraged. Standardizing timing of interventions in surgical emergencies will promote clinical investigation as well as a commitment by administrative authorities to proper operating theater provision for acute care surgery.

## Introduction

Triage is the process of defining the priority of patients’ management according to the severity of their disease process and clinical condition. This process is of paramount importance when resources are insufficient for patient demand and when medical team availability is lacking. Triage is also initiated to avoid resource exhaustion. The process ensures proper care in a timely manner for the sickest. The main principle is saving lives. The outcome and grading of patients is frequently the result of clinical assessment and physiological findings. Modern approaches to triage are scientific and systematic and some are algorithm-based. As triage concepts have become more sophisticated, software and hardware decision support products have evolved to guide caregivers in both hospitals and in the field. Triage is practiced in mass casualty incidents and its rationale is accepted worldwide. Such systems should also be implemented for the care of surgical emergencies other than injury related. In these cases, the concept of triage is especially important for managing multiple patients with diverse needs. Currently, timing emergency surgery is a matter of individual interpretation of the common adjectives used in the literature to express the degree that surgery is- emergent, prompt, early, urgent, expeditious and immediate. Further research on the proper timing of surgery will enable the translation of these adjectives to a more consistent time frame commitment. Evidence based data to support rigorous triage of non-traumatic surgical emergencies should be established and triage policies developed and implemented worldwide. Until this objective will evolve certain agreements on mechanism and principles of triage of emergency surgeries can be delineated.

This manuscript is an overview of the challenges of timing emergency, non- traumatic operations, with recommendations based on an international expert opinion survey (TACS study).

### Review of literature and expert opinions

Acute care surgery requires punctual evaluation and early intervention, usually for diseases of short duration. The notion that expeditious management of acute surgical diseases is the appropriate strategy is based on the knowledge that delaying treatment may increase the risks of adverse outcomes. This study was approved by the ethical committee of the Rambam Health Care Center.

Most non-traumatized surgical patients present to the emergency department with one of three leading complaints: 1. abdominal or groin pain, 2. gastrointestinal bleeding 3. soft tissue infection. After thorough investigation, most of these clinical patterns evolve into unambiguous diagnoses. Some of the clinical patterns that represent acute surgical disease are managed by emergency surgery. Moreover, in certain situations, only surgery leads to proper diagnosis. Other situations require further nonsurgical investigation, and may be treated sufficiently by conservative management. Deferring surgery to daytime hours is appropriate in certain situations. On the other hand, inappropriate delaying of surgery may result in further contamination of the abdominal cavity (perforation of duodenal ulcer, perforated diverticulitis) or perforation of an inflamed organ (appendix) if left untreated. Soft tissue infections (perianal abscess, gluteal abscess) may progress to soft tissue gangrene if treatment is postponed, especially in patients who suffer co- morbidities, such as diabetes mellitus. Delaying treatment in a patient with mesenteric vascular insult may result in frank bowel necrosis or in extension of the ischemia, resulting in a protracted postoperative course and eventually death.

Papandria et al. found that delay to appendectomy is associated with increased perforation rates in children and adults [1]. This finding concurs with previous studies and with the conventional progressive pathophysiologic appendicitis model. On the other hand, Eko et al. found that timing of surgery for acute appendicitis did not affect the incidence of complications including perforation. However, in that study, delay in surgical consultation and treatment was associated with increased length of hospital stay and increased hospital costs. The investigators concluded that optimal timing of appendectomy for uncomplicated acute appendicitis appears to be within 18 hours of emergency department presentation [2]. In contrast, Abou Nukta et al. claimed that delaying appendectomy for 12–24 hours does not have a significant effect on perforation rate, operative time or length of hospital stay [3]. In an attempt to clarify the risk of surgical delay in acute appendicitis the ACS National Surgical Quality Improvement Program (ACS NSQIP) database was reviewed [4]. The primary outcomes were 30-day overall morbidity and 30-day serious morbidity and mortality. Patients who underwent appendectomy more than 12 hours after hospital admissions fared no worse than those who underwent surgery less than six hours from the time of admission. These data support the scheduling of appendectomies for the earliest, yet most suitable time for the surgeon and for proper hospital resource utilization and expenditure, which is usually in the morning. Several studies have addressed the optimal time for surgical intervention in acute cholecystitis [5] and diverticulitis [6]. Pakula et al. recently showed that delaying surgery in patients diagnosed with necrotizing fasciitis did not increase the risk of mortality [7]. Chao et al. [8] echoed Pakuals’ observation indicating that timing of surgery (within 12 hours of admission) didn’t impact outcome of patients admitted for Vibrio- vulnifics- related necrotizing fasciitis. Korkut et al. [9] on the contrary claim that the interval from the onset of clinical symptoms to the initial surgical intervention seems to be the most important prognostic factor with a significant impact on outcome of patients with Fournier’s gangrene.

The objective of the management of acute surgical diseases is to save lives by controlling bleeding or contamination, or by improving organ perfusion. This objective obligates the need for strong commitment and effective mechanisms for prioritizing patient management according to physiological and clinical parameters. Resource availability along patient physiological and clinical parameters in the acute care arena justifies the development of triage tools and agreed criteria for proper timing of emergency operations. Most studies on timing of surgery have investigated delays in operations. This may reflect problems of resource availability, and indicate a need for all parties involved in surgical emergencies, both caregivers and their employers, to commit to high quality of care. Convenience for caregivers or administrators should not override patient safety. Investigations of the influence on patient outcomes of surgical delays due to constraints of resource utilization, must consider the availability of operating theaters at any given time.

Despite the widespread adoption of acute care surgery as a specialty among other surgical professions, the implementation, standardization and development of this discipline vary considerably among medical centers [10]. The World Society for Emergency Surgery (WSES) conducted an international expert opinion panel (TACS). Members of this panel were asked to fill a questionnaire that included information on their acute care service in regard to operating room availability for emergency cases, as well as hospital case load (Table 1). Of the 88 WSES expert panel members receiving the survey, 43 (48.6%) responded. Of the respondents, 79% indicated that a dedicated acute care surgery service operates in their hospital and 71.9% activate a dedicated operating theater (1–3, 72.9%). From this survey it appears that institutions around the world have adopted the concept of acute care services, and established appropriate use of their facilities. Nevertheless, only 51.2% of the respondents indicated that triage of surgical emergencies is performed by a surgeon.

**Table 1:** International survey on ACS systems

	***n- 43(%)***
***Number of Hospital Beds***	
< 250	2 (4.8)
250–500	9 (21.4)
500–750	10 (23.8)
750–1000	10 (23.8)
> 1000	11 (26.2)
***Number of General Surgery Cases***	
*< 1000*	27 (62.8)
*1000–2000*	8 (18.6)
*2000–3000*	4 (9.3)
*> 3000*	3 [7]
***Dedicated Acute Care Service***	34 (79.1)
***Dedicated OR for Emergency cases***	34 (79.1)
***Activated OR for Emergency Cases***	
*1–3*	31 (72.9)
*3–6*	8 (18.6)
*7–10*	4 (9.3)
***Triage system for Emergency Cases***	10 (23.3)
***Does Color Coding is Suitable for Triage of Emergency Cases***	31 (88.6)
***Who is Your Triage Officer***	
*General Surgeon*	20 (46.5)
*Anesthesiologists*	18 (41.9)
*Acute Care Surgeon*	2 (4.7)
*Anesthesiologist + General Surgeon*	1 (2.3)
*Casualty Medical Officer*	1 (2.3)
*None*	1 (2.3)
*OR – Operating Room*	

In addition, 41.9% reported that an anesthesiologist is assigned as triage officer at their institution; 23.3% indicated that they already activate a triage system in their hospitals for general surgery emergencies, and 88.6% agreed to the need for such arrangement (Table 1).

When an injured patient presents to the Emergency Department with hemodynamic instability due to a traumatized bleeding spleen, the need for immediate surgery is apparent, and the healthcare team prepares in an almost routine fashion to deliver care and surgical intervention without delay. This is well-accepted, taught and practiced worldwide, and is the result of long standing efforts in education and proper trauma system organization. The simultaneous presentation of many injured patients in need of surgery prompts initiation of triage criteria. After establishing patent airway and ensuring normal breathing mechanism, hemodynamic instability is assigned first priority [11]. Triage criteria for the management of the injured are based on extensive experience gained during war times, and on research, knowledge acquisition and observations by surgeons who dedicated their career to the management of the wounded.

In the management of mass casualty incident, patients are triaged using a color coding system [12]. Prioritizing care of injured patients in need of surgical interventions is based on the same color coding system. This system was developed from the experience of military and civilian mass casualty incidents. Preparedness is crucial for successful treatment of the medical aspect of mass casualty incidents [13].

Hospital color codes alert staff to various emergencies. They convey common and repetitive language and are essential for the distribution of rapid, comprehensible and well-accepted information. We propose that the use of a color coding system to triage emergency surgery cases may help to reduce information loss and time spent on conferring with other caregivers regarding scheduling of emergency operations. Color coding will enable the use of common and standardized language among the members of the surgical team, and other acute care surgical disciplines and will allow proper documentation and improved quality of care.

Treating surgical emergency non- traumatized patients involves the same principles used in the management of the traumatized. Team availability and preparedness, prompt effort at diagnosis and early initiation of management protocols are the hallmarks of the acute care surgery approach for the most severely ill. Immediate availability of resources is essential. Triage concepts and color coding should therefore be adopted in the management of surgical emergencies as well.

In a busy Emergency Department with an influx of patients in need for early intervention, assigning patients to surgery in a “timely manner” is mastery. Triage criteria based on data and knowledge of disease processes need to be set forward for non- traumatic surgical emergencies. Setting proper time frames will promote the establishment of international standards, the initiation of worldwide research and the development of acute care services by national authorities and hospital management administrations. Triage criteria for acute surgical diseases should include simple hemodynamic and clinical data. These criteria would direct the acute surgical teams to properly tag each patient to the timing of surgery. While committing to the time frame set forward for managing patients with surgical emergencies, appropriate steps should be undertaken for optimizing patient physiological status alongside antibiotics administration and pain control during the wait for surgery. Acute Care Surgeons must decide on a proper time frame for the management of their patients, and to commit the medical system to such time frame. This commitment is essential especially in busy medical centers where the Emergency Department is crowded with patients in need of surgery, yet lacking availability of operating theaters.

### Classification system

Considering the above (TACS study and current literature), the following categories could be incorporated into a triage system of acute care surgery cases as follows:

***Immediate*-** implies an extreme or markedly decompensated physiological state, usually resulting from bleeding. This is rare in non- traumatic surgical emergencies, and for most bleeding patients initial resuscitative measures will enable further evaluation, diagnosis and even non-operative treatment. Active intra peritoneal bleeding due to a ruptured visceral aneurysm, a ruptured spleen due to hematological disorder with bleeding are examples of a condition that requires immediate surgery. In this category, life or tissue loss is imminent.

***Within an hour from diagnosis***- implies signs and symptoms of vascular compromise: incarcerated hernia with bowel entrapment, mesenteric vascular occlusion, or limb ischemia. Diffuse peritonitis due to uncontained hollow viscus perforation is another example of a condition that requires surgery “within an hour” as is the presence of necrotizing fasciitis with sepsis. Patients diagnosed with these pathologies need to be adequately resuscitated and managed while undergoing further diagnoses and other steps toward safe surgery. Physiologically, patients may have signs of sepsis or mild to moderate organ dysfunction requiring rapid resuscitation without delaying surgical intervention. In most cases, tissue loss is imminent.

***Within 6 hours from diagnosis***- implies localized peritonitis or soft tissue infection in need of surgery, but not a physiological state that entails spreading or progression of the disease process. These pathologies have the potential to evolve to more serious conditions if surgery is delayed. Antibiotic treatment and fluid administration should be initiated immediately upon diagnosis and repeat examination carried out while waiting for surgery.

***Within 12 hours from diagnosis***- implies a need of surgery, though evidence- based knowledge indicates that postponing surgery while under medical treatment does not lead to clinical deterioration. As an example, delay in treatment of acute appendicitis has been shown to have no deleterious effect on outcomes.

***Within 24 or 48 hour from diagnosis***- Suggests that intervention is indicated and the process may progress and worsen the morbidity of the operation. Examples include cholecystitis and thoracic empyema. The classification also applies to patients who were operated under emergency, and re-laparotomy was decided upon during the index procedure for peritoneal cavity rinsing or for assessment of bowel perfusion and viability. These principals need to be adopted, understood and appreciated by all personnel involved in the treatment of patients with surgical emergencies.

### Timing of surgical intervention

Prompt, early, urgent, expeditious, immediate, and emergency are common adjectives used in the medical literature to describe the need for surgery “in a timely manner”. The literature lacks evidence based data on proper timing of emergency surgery. Definitions of Time To Surgery (TTS), Ideal Time To Surgery (iTTS) and Actual Time To Surgery (aTTS) should therefore evolve and be standard for further discussions. Launching a triage system for non- trauma surgical emergencies will ensure that time to surgery (TTS) develops into a quality improvement tool. Actual TTS (aTTS, real time waiting for surgery) can be compared to the time assigned for each pathology by expert opinion, consistent with data from current literature (ideal time to surgery, iTTS). The ratio aTTS/iTTS will reflect efficiency and should be used for quality assessment. A ratio of ≤ 1 indicates compliance with standards for timing of surgery and a ratio >1 indicates that surgery was delayed. Delaying surgery from the time set by the acute surgical care team and determined by the triage system will be a matter for further quality improvement measures.

A reproducible tagging mechanism for acute surgical emergencies will clarify the vague adjectives commonly used to describe the urgency of “emergent” surgery. This will enable definitions of worldwide criteria for the timing of emergency surgery. When dealing with surgical emergencies, descriptive words for “timely surgery” should be substituted with unambiguous and reproducible time frames. This needs to be scrutinized, tested and validated on a worldwide scale.

**Figure 1:**
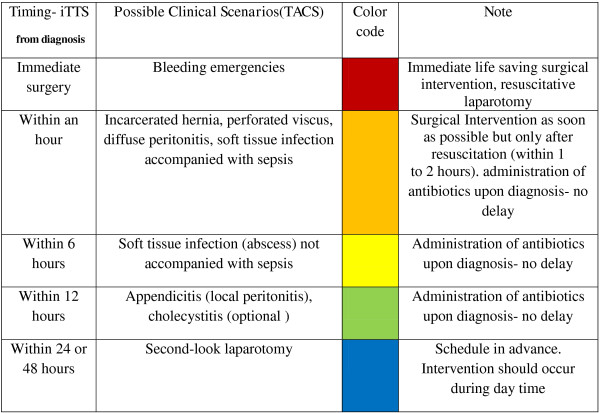
Proposed Ideal Time to Surgery (iTTS) and color coding.

In an effort to understand current occurrence in acute care of surgical emergencies and common practices of emergency surgery scheduling, WSES panel experts were asked to assign iTTS to a number of common surgical emergencies - acute appendicitis, incarcerated inguinal hernia, mesenteric ischemia, perforated duodenal ulcer and peri- anal abscess. The results are summarized in Table 2. The TACS study identified high agreement among responders regarding the time frame presented for common surgical emergencies. Although the data presented in the table does not concur with current views in the literature regarding some of the clinical entities surveyed, this may reflect availability of operating theaters in some of the institutions participating in the study. In most institutions, scheduling of unplanned is a matter of dialogue and negotiation where dedicated operating theaters are not assigned for surgical emergencies. The discrepancy revealed in iTTS assessment between TACS respondents and the current literature, e.g. timing of appendectomy [3] and cholecystectomy [5], indicate that further studies are needed to establish iTTS for surgical emergencies. Until this is accomplished a certain frame of iTTS can be proposed and implemented as an interim guideline for the timing of surgical interventions in surgical emergencies as proposed in Figure 1.

**Table 2:** Expert opinion on timing of surgery in common surgical emergencies

	***n-43(%)***
***Immediate Surgery***	
*Mesenteric Event*	37 (86)
*Evisceration*	27 (62.8)
*Hemodynamic Instability due to bleeding*	42 (97.7)
***Surgery Within an Hour***	
*Incarcerated Hernia*	35 (83.3)
*Perforated Viscus*	35 (83.3)
Necrotizing Fasciitis*	34 (79.1)
***Surgery Within 6 Hours***	
*Soft Tissue Infection (Abscess)*	37 (86)
Appendicitis*	36 (83.7)
Cholecystitis*	29 (67.4)
***Surgery Within 24–48 Hours***	
*Second Look Laparotomy*	41 (95.3)

*expert opinion not in aptness with current literature.

The National Confidential Enquiry into Patient Outcome and Death (NCEPOD) in the United Kingdom classifies interventions as immediate, urgent, expedited and elective [14]. For each of these categories, the respective target times to theatre from decision to operate is within minutes, hours, days or planned. There is general agreement that cases requiring immediate attention will be triaged before less urgent cases. Cases classified between these two groups raise the greatest debate in terms of patient priority. A standardized system for categorizing clinical urgency would be most beneficial to these types of surgical cases. It is clear from the TACS study and from other available guidelines [14] that iTTS is a matter of consensus among care providers based on clinical data.

iTTS needs further scrutinizing in regard to each and every surgical emergency and further investigation on the impact of actual time to surgery (aTTS) on outcomes. The goal is to establish evidence-based and feasible triage criteria for appropriate timing of operation in surgical emergencies.

Recommendations:

1. We recommend adopting a color-triage system for acute surgical emergencies.

2. We suggest that each medical institution should examine its aTTS and compare it to the iTTS proposed in this paper. This will facilitate the conduct and comparison of international research, and will ease adoption of triage protocols for surgical emergencies.

3. We recommend using the aTTS/iTTS ratio as a quality improvement tool and as an international index for comparison in future research.

4. We recommend that further studies on appropriate timing of emergency surgeries be initiated, and that the findings be implemented in more refined triage systems.

## Conclusions

Accumulating evidence on the impact of delaying emergency surgical intervention on patient outcomes challenges common knowledge and intuitive paradigms held by acute care surgeons. The need for prospective multi-institutional studies on the appropriate timing of operations for surgical emergencies has become clear.

## Competing interests

The authors declare that they have no competing interests.
